# Mitochondrial complex I subunit deficiency promotes pancreatic α-cell proliferation

**DOI:** 10.1016/j.molmet.2022.101489

**Published:** 2022-04-04

**Authors:** Xuefei Yu, Catherine Arden, Rolando Berlinguer-Palmini, Chun Chen, Carla Bradshaw, Anna LM Smith, Julia Whitehall, Michael White, Scott Anderson, Nicole Kattner, James Shaw, Doug Turnbull, Laura C Greaves, Mark Walker

**Affiliations:** 1 Diabetes Research Group, Translational and Clinical Research Institute, Newcastle University, Newcastle upon Tyne, UK; 2 The BioImaging Unit, Newcastle University, Newcastle upon Tyne, UK; 3 Wellcome Centre for Mitochondrial Research, Translational and Clinical Research Institute, Newcastle University, Newcastle upon Tyne, UK; 4 Wellcome Centre for Mitochondrial Research, Biosciences Institute, Newcastle University, Newcastle upon Tyne, UK

**Keywords:** Mitochondria, mtDNA, mtDNA mutator mice, Pancreatic islets

## Abstract

**Objective:**

There is strong evidence that mitochondrial DNA mutations and mitochondrial dysfunction play a role in diabetes pathogenesis. The homozygous knock-in mtDNA mutator mouse is a model of premature aging due to the accumulation of mitochondrial DNA mutations. We used this mouse model to investigate the relationship between mitochondrial subunit expression and pancreatic islet cell composition.

**Methods:**

Quadruple immunofluorescence was used to quantify mitochondrial subunit expression (complex I and IV) and cell composition in pancreatic islets from mitochondrial DNA mutator mice (*PolgA*^*mut/mut*^) and control C57BL/6 mice at 12 and 44 weeks of age.

**Results:**

Mitochondrial complex I subunit expression was decreased in islets from 12 week *PolgA*^*mut/mut*^ mice. This complex I deficiency persisted with age and was associated with decreased insulin staining intensity at 44 weeks. Complex I deficiency was greater in α-cells compared with β-cells in islets from 44 week *PolgA*^*mut/mut*^ mice. Islet cell composition was normal in 12 week *PolgA*^*mut/mut*^ mice, but the β: α cell ratio was decreased in islets from 44 week *PolgA*^*mut/mut*^ mice. This was due to an increase in α-cell number linked to an increase in α-cell proliferation.

**Conclusion:**

Complex I deficiency promotes α-cell proliferation and alters islet cell composition.

## Highlights

• Decreased islet complex I expression was established in *PolgA*^*mut/mut*^ mice at 12 weeks.
• Decreased islet complex I expression was associated with increased pancreatic α-cell number in *PolgA*^*mut/mut*^ mice at 44 weeks.
• Increased α-cell proliferation contributes to the increased α-cell number.

## 1 Introduction

The prevalence of type 2 diabetes increases with age [[1], [2], [3]], which is in part the result of a progressive decline in pancreatic β-cell function [4] and insulin secretion [5]. Additionally there is evidence of altered pancreatic islet cell composition in type 2 diabetes, with a decrease in the β:α cell ratio [6,7]. The molecular mechanisms driving these changes remain to be fully elucidated, but there is growing evidence that mitochondrial dysfunction plays a role in diabetes pathogenesis [8].

Mitochondria are semi-autonomous organelles which produce ATP through oxidative phosphorylation (OXPHOS). Mitochondrial DNA (mtDNA) is present in multiple copies within individual cells and the copy number is correlated with the cell's energy demand. The multi-copy nature of mtDNA means that most mtDNA mutations are functionally recessive – a significant proportion of molecules must be mutated in order for a biochemical defect to manifest [9]. Germline mtDNA mutations are a cause of human metabolic disease, including diabetes [10], and somatic mtDNA mutations have been shown to accumulate in a number of aging human tissues e.g. heart, skeletal muscle, gut, liver and brain [11].

There is strong evidence that mtDNA mutations and mitochondrial dysfunction play a role in diabetes pathogenesis. Maternally inherited diabetes and deafness (MIDD) is the most common manifestation of the m.3243A > G mutation [12] with particularly high levels of the mutations detected in islets [13] and a mosaic pattern of pancreatic β-cell loss, probably due to low tolerance of mitochondrial dysfunction in these cells [14]. In addition, β-cell specific knock-out (KO) of the mitochondrial transcription factor alpha (TFAM) results in mtDNA depletion and severe OXPHOS deficiency. This leads to reduced insulin secretion followed by β-cell loss recapitulating the β-cell pathology seen in mitochondrial diabetes [15]. An alternative mouse model in which to investigate the role of mitochondrial dysfunction in diabetes pathogenesis is the mitochondrial ‘mutator mouse’ (*PolgA*^*mut/mut*^). These mice have a D257A amino acid change in the proof-reading domain of the mtDNA polymerase gamma, and consequently accumulate high levels of mtDNA mutations over time, resulting in reduced lifespan and a premature aging phenotype [16]. Our primary aim was to use this mouse model to investigate the relationship between mitochondrial subunit expression and pancreatic islet cell composition.

## 2 Material and methods

### 2.1 Animals

Mitochondrial mutator mice (*PolgA*^*mut/mut*^) used in our study were originally generated by inserting a knock-in mutation (D257A) in the second endonuclease proofreading domain of the PolgA catalytic subunit of the mtDNA polymerase, on a C57BL/6J background. Wild type mice (*PolgA*^*+/+*^) (from the previous generation to avoid the transmission of maternally inherited mtDNA mutations [17]) were used as controls. All mice used in our experiments were group housed regardless of their genotypes (3–5 mice per cage) under a 12hr light/dark cycle at room temperature (25ᵒC, RT). *PolgA*^*mut/mut*^ mice aged 12 weeks (n = 4) and *PolgA*^*mut/mut*^ mice aged 44 weeks (n = 5) were grouped as young and old experimental groups respectively. *PolgA*^*+/+*^mice aged 12 weeks (n = 4) and *PolgA*^*+/+*^mice aged 44 weeks (n = 5) were assigned to each experimental group as a control for comparison. Additional *PolgA*^*mut/mut*^ mice (n = 5) and age-matched *PolgA*^*+/+*^ mice (n = 5) were used for end-point blood glucose measurement at 44 weeks. Mouse details are reported in Supplementary Table 1. All animal experiments were conducted in compliance with the UK Home Office (PPL P3052AD70) and the Newcastle University Animal Welfare Ethical Review Board (AWERB 425).

### 2.2 Tissue preparation

Mice were sacrificed by cervical dislocation and the whole pancreas was harvested. Half of the pancreas was fixed in 10% neutral buffered formalin for 24 h at room temperature followed by standard tissue processing and paraffin embedding, and the other snap frozen in liquid nitrogen and stored at −80 °C.

### 2.3 Mitochondrial quadruple immunofluorescence

4 μm sections of formalin-fixed, paraffin-embedded tissues were subjected to quadruple immunofluorescence essentially as previously described [18]. 4 μm sections were de-paraffinised and rehydrated as standard. Antigen retrieval was performed by pressure-cooking in 1 mM EDTA pH8.0, followed by incubation in primary antibodies (information is in Supplementary Table 2) overnight at 4 °C. Sections were washed in TBST and incubated in secondary antibodies (Supplementary Table 3) for 2 h at room temperature. Sections were then washed in TBST and mounted using Prolong Gold Antifade Mountant (Invitrogen).

### 2.4 Mitochondrial quadruple immunofluorescence imaging and analysis

Imaging was performed using a Nikon A1 laser scanning confocal microscope using 405, 488, 546 and 647 nm excitation lines. 50 islets were studied for each mouse. In order to quantitatively compare the mitochondrial subunit protein levels between groups, the laser power remained constant across all experimental groups. Images were analysed using NIS-element analysis software. Each islet was selected as a region of interest (ROI) and the fluorophore mean intensity values recorded for each channel. Values were background corrected by subtracting the mean intensity of the no primary control (NPC) from the ROI mean to exclude background fluorescence. Background corrected intensity values were log transformed to normalize the data. Statistical analysis was performed using in house software (available at http://mito.ncl.ac.uk/immuno/) as previously described [18]. In brief, a control group was generated by random sampling of an equal number of intensity values from the islets of the control groups for each individual analysis (see results for details). Linear regressions of MTCO1 and NDUFB8 values (dependent variables) against Tomm20 values (independent variable) were performed, validated to ensure the residuals of the regression were normally distributed, and the standard error of estimate determined. This allowed the estimate of deviation of MTCO1 and NDUFB8 levels in each islet from the predicted level according to the Tomm20 level. The parameters (mean and standard deviation) describing the distribution of Tomm20 in the control population was determined as well as parameters describing the linear relationship between MTCO1 versus Tomm20 and NDUFB8 versus Tomm20. Z-scores were determined for Tomm20 (Tomm20_Z), as well for NDUFB8 (NDUFB8_Z) and MTCO1 (MTCO1_Z) based on the expected level of each according to the Tomm20 level. Negative z-scores indicate lower levels of each protein than expected based on the control population, and vice versa for high z-scores.

### 2.5 Islet cell composition immunoflourescent labelling

4 μm sections were de-paraffinised and rehydrated as standard. Antigen retrieval was performed by pressure-cooking in 10 mM Sodium Citrate (Sigma) buffer (pH = 6.0). Sections were then incubated with 20% FBS (in PBS) for 1 h at room temperature and then incubated overnight with antibodies against islet endocrine hormones insulin and glucagon, and cell proliferation protein Ki67 at 4 °C (Supplementary Table 2). On the following day, sections were washed in PBS and then incubated for 2 h at room temperature with the secondary antibody cocktail (Supplementary Table 3). After washing in PBS, sections were then incubated for 15 min at room temperature in DAPI (Bio-Rad, 1: 50,000 in PBS). Slides were then mounted using vectashield mounting medium with DAPI (Vector laboratory).

### 2.6 Islet cell imaging and analysis

Imaging was performed using a Nikon A1 laser scanning confocal microscope using 405, 488, 546 and 647 nm excitation channels. 25 islets were studied per mouse, with the optimal laser power set on an individual islet basis to allow accurate detection of alpha and beta cells within each islet. NIS-element analysis software was used to detect the nucleus (405 nm), Ki67 (488 nm), α-cells (glucagon, 546 nm) and β-cells (insulin, 647 nm). Co-localised labelling of the nucleus and glucagon channels identified α-cells and β-cells were identified by co-localised labelling of the nucleus and insulin markers. Bihormonal (glucagon and insulin positive) cells were identified by co-localised labelling of the insulin, glucagon and nucleus markers. The numbers of cells in each category per islet was counted. The islet size was measured by drawing around the periphery of each islet. Absolute α- and β-cell number, β: α ratio, whole islet cell number, islet size, α- and β-cell percentage and Ki67 (+) islets were counted.

### 2.7 (TdT)-mediated dUTP nick end labelling (TUNEL) apoptosis assay

Apoptotic cells were detected using an In Situ Cell Death Detection kit (Merck, 11684817910) as per the standard manufacturer's protocol with the following exceptions: the enzyme solution was diluted 1:40 in TUNEL dilution buffer (Merck, 11966006001) and the convertor-POD was diluted 1:2 in PBS. Slides were imaged using the Aperio virtual pathology system (Leica Microsystems, UK) and analysed using Aperio Imagescope v12.4.

### 2.8 Blood glucose testing

44 week *PolgA*^*mut/mut*^ and *PolgA*^*+/+*^ mice were weighed and then euthanised and terminal blood collected. Alphatrak glucometer (Animed) was used to determine endpoint blood glucose concentrations as per the manufacturer's standard protocol

### 2.9 Statistical analysis

Statistical analyses were performed by using Graphpad prism software (v.8.3). All data were checked for normality and unpaired t-tests used to make comparisons between groups. The islet data are presented as mean ± 95 %CI. The metabolic and anthropometric data are presented as mean ± SEM. The Mann Whitney test was used for non-normal data, and the data presented as median (interquartile range). P < 0.05 was the threshold for statistical significance.

## 3 Results

### 3.1 Decreased complex I protein levels are detectable from 12 weeks of age in *PolgA*^*mut/mut*^ islets and persist with advancing age

**Figure 1:**
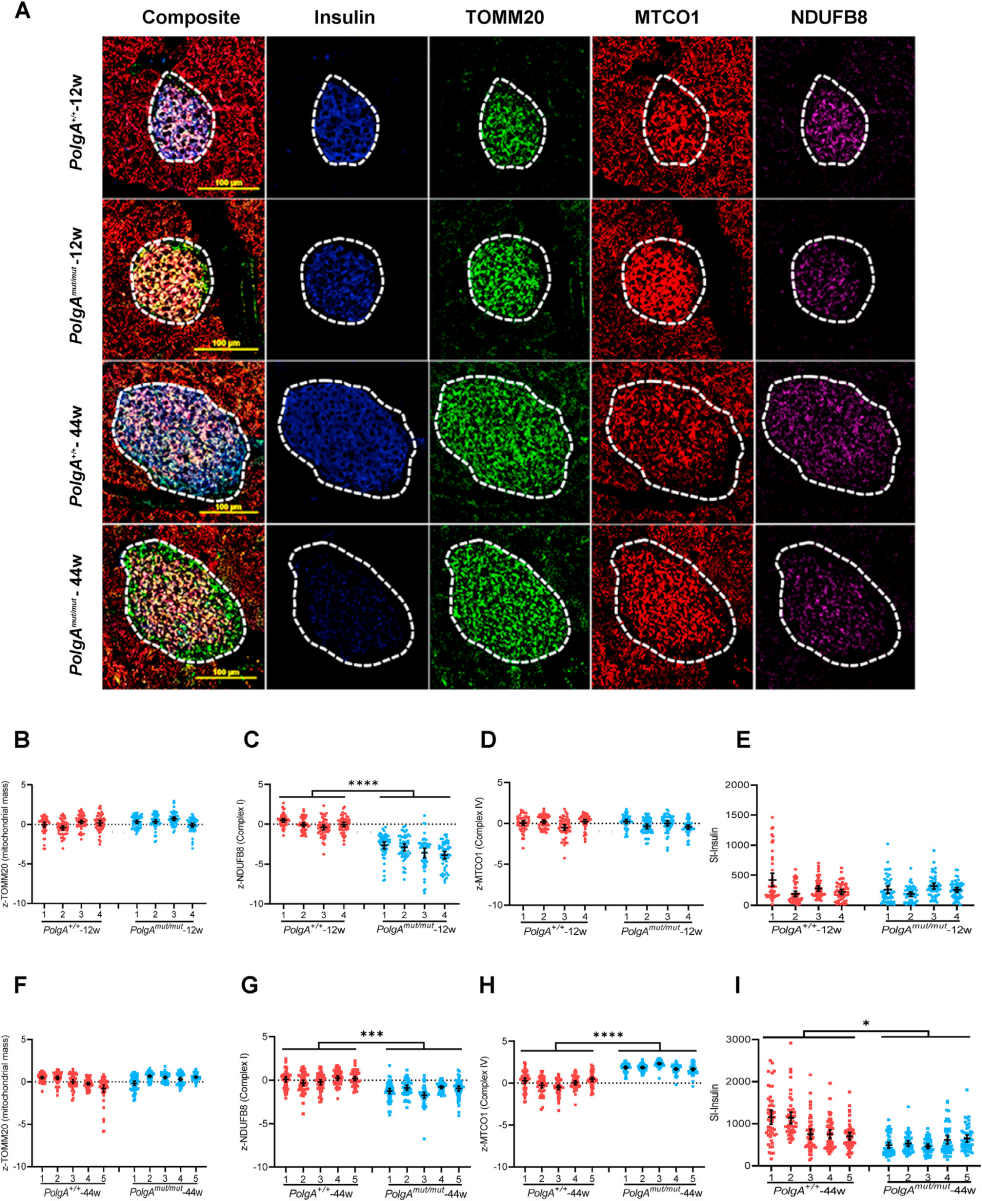
**Decreased complex I expression is present in *PolgA*^*mut/mut*^ islets from 12 week mice and persists with advancing age**. (A): Representative immunofluorescent panel showing labelling of mitochondrial proteins in pancreatic islets from 12 week to 44 week mice. Scale bar, 100 μm. The dashed white line marks the islet perimeter. (B–E): Quantitative analysis of levels of TOMM20 (mitochondrial mass) (B), NDUFB8 (Complex I) (C) MTCO1 (Complex IV) (D), and insulin (E), in islets from 12 week *PolgA*^*mut/mut*^ and *PolgA*^+/+^ mice. Data are presented as Z-scores relative to the *PolgA*^*+/+*^ mice, n = 4 mice per group. (F–I): Quantitative analysis of levels of TOMM20 (F), NDUFB8 (G) MTCO1 (H), and insulin (I), in islets from 44 week *PolgA*^*mut/mut*^ and *PolgA*^+/+^ mice. Data are presented as Z-scores relative to the *PolgA*^*+/+*^ mice, n = 5 mice per group. For panels B–I each point represents an individual islet (n = 50 per mouse). Data are presented as mean ± 95% CI. Unpaired t-test. ∗P < 0.05. ∗∗∗P < 0.001. ∗∗∗∗*P* < 0.0001.

We investigated the levels of mitochondrial OXPHOS subunit proteins in islets from *PolgA*^*mut/mut*^ and *PolgA*^*+/+*^ mice at 12 and 44 weeks using quadruple immunofluorescence. Individual islets were labelled for; complex I (NDUFB8), complex IV (MTCO1), mitochondrial mass (TOMM20) and insulin (Figure 1A). Intensity of insulin labelling was quantified per islet. Levels of NDUFB8 and MTCO1 per islet were quantified and normalised to TOMM20. This assay was previously validated in human patients with clinically diagnosed primary mitochondrial disease, where it was shown to accurately detect individual cells with complex I and complex IV deficiency caused by either mtDNA or nuclear DNA mutations [18]. It has also been used to confirm complex I and IV deficiency in the *PolgA*^*mut/mut*^ mouse colon and small intestine [19,20]. All data are presented as Z-scores compared with either age-matched *PolgA*^*+/+*^ mice for between genotype comparisons or between 12 week and 44 week old mice for within-genotype comparisons.

We first compared the levels of OXPHOS proteins in *PolgA*^*mut/mut*^ and *PolgA*^*+/+*^ mice at 12 weeks of age (Figure 1B–D). There was a significant reduction in the levels of the NDUFB8 subunit of complex I in pancreatic islets from *PolgA*^*mut/mut*^ mice compared with age-matched *PolgA*^*+/+*^ mice (Figure 1C). No significant differences in the levels of complex IV were detected (Figure 1D), nor was there evidence of any change in mitochondrial mass (Figure 1B). Next we compared the OXPHOS protein expression pattern at 44 weeks of age (Figure 1F–H). Levels of complex I remained significantly lower in *PolgA*^*mut/mut*^ mouse islets compared with age-matched *PolgA*^*+/+*^ controls (Figure 1G), however there was a significant increase in levels of complex IV (Figure 1H). There was no difference in mitochondrial mass between the genotypes (Figure 1F). This was also corroborated by analysis of mtDNA copy number which showed no significant differences between the genotypes at 44 weeks (Supplementary Fig. 1).

We next studied the changes between 12 and 44 weeks of age within each genotype. In *PolgA*^*+/+*^ mice there was a significant decrease in the levels of complex IV between 12 and 44 weeks (Supplementary Fig. 2C) but mitochondrial mass and complex I remained unchanged (Supplementary Figs. 2A and B). Islets from *PolgA*^*mut/mut*^ mice showed no age-related changes in either complex I or IV (Supplementary Figs. 2E and F) but did show a significant increase in levels of mitochondrial mass (Supplementary Fig. 2D).

### 3.2 Complex I subunit deficiency is associated with decreased insulin levels, increased α-cell number, and altered islet cell composition in 44 week old *PolgA*^*mut/mut*^ mice

**Figure 2:**
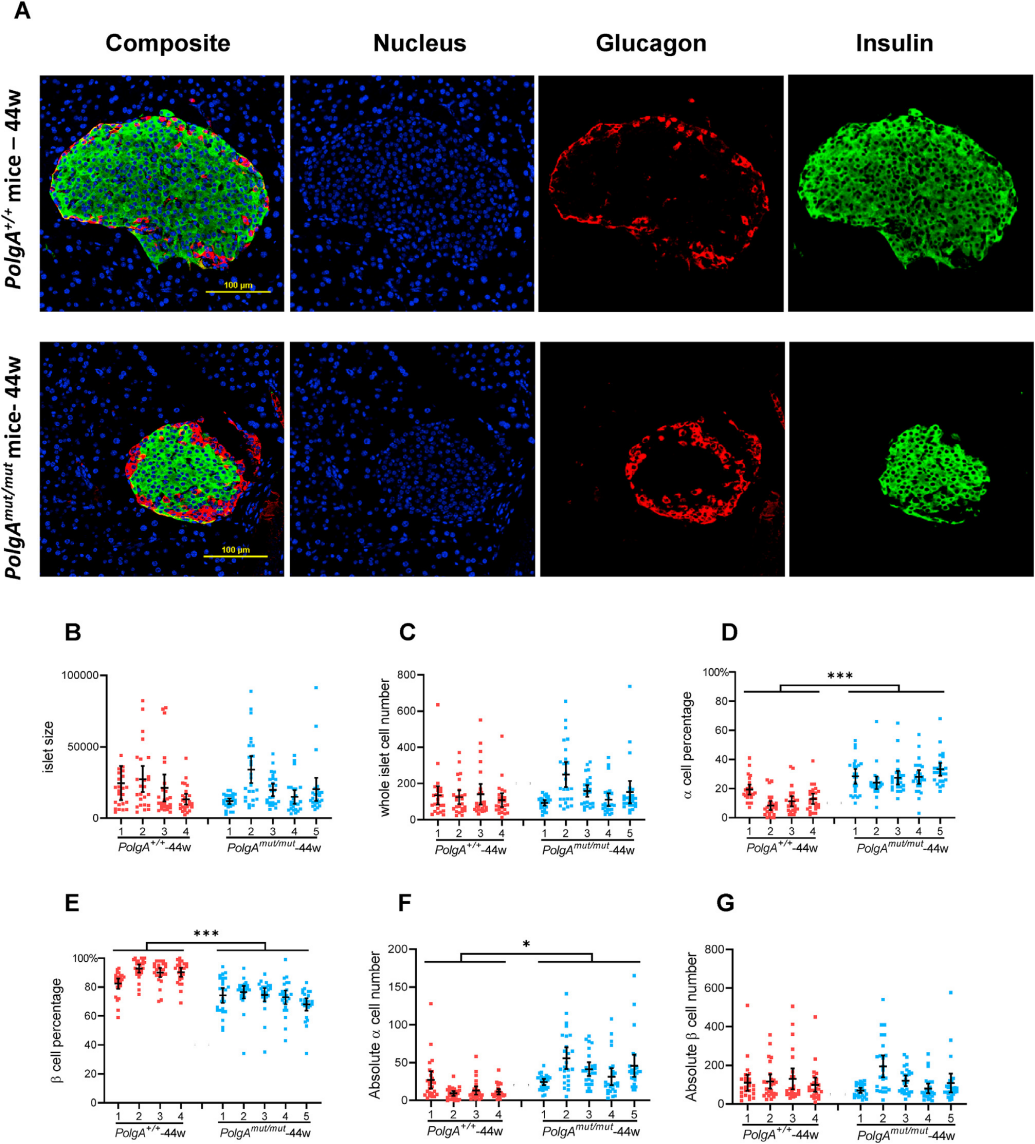
**Islets from 44 week *PolgA*^*mut/mut*^ mice have increased numbers of α cells resulting in altered islet cell composition** (A): Representative immunofluorescent panel showing labelling of nuclei (DAPI), α-cells (Glucagon) and β-cells (insulin) in islets of 44 week *PolgA*^*mut/mut*^ and *PolgA*^*+/+*^ mice. Scale bar, 100 μm. (B–G): Quantification of the islet size (B), total cell number (C), α-cell percentage (D), β-cell percentage (E), absolute α-cell number (F), and absolute β-cell number (G) in 44 week *PolgA*^+/+^ (n = 4) and *PolgA*^*mut/mut*^ (n = 5) mice. Each point represents one islet (n = 25 for each mouse). Data are presented as mean ± 95% CI. Unpaired t-test. ∗*P* < 0.05. ∗∗∗*P* < 0.001. Microscope settings were individually adjusted according to each islet.

We next investigated the effect of mitochondrial complex I deficiency on levels of insulin and islet cell composition. The insulin labelling intensity was measured by using quantitative immunofluorescence as described above (Figure 1E,I). For the islet cell composition study, separate sections were labelled using antibodies against insulin (to label β-cells), glucagon (to label α-cells) and counterstained in DAPI (to label all cell nuclei). Protein levels were not quantified, but scored as present or absent per cell nuclei. Confocal laser power was optimised per islet for optimal identification of positive or negative cells. As a consequence, insulin staining in the 2 illustrative islets shown in Figure 2A should not be taken as representative of the protein levels.

Comparisons were first made between *PolgA*^*mut/mut*^ and *PolgA*^*+/+*^ genotypes. At 12 weeks of age, even though there was a significant complex I defect in *PolgA*^*mut/mut*^ mice, there were no significant differences in islet size, the absolute numbers or proportions of α-cells or β-cells (Supplementary Fig. 3), or insulin staining intensity (and Figure 1A,E) compared with age-matched *PolgA*^*+/+*^ mice. However, at 44 weeks of age, despite no significant differences in islet size (both area and total cell number) (Figure 2B,C), significant differences in the relative proportions of α-cells and β-cells were detected in *PolgA*^*mut/mut*^ islets compared with *PolgA*^*+/+*^ islets. There was a significantly higher percentage of α-cells and a significantly lower percentage of β-cells (Figure 2D,E). These changes were driven by a significant increase in the absolute number of α-cells in islets from *PolgA*^*mut/mut*^ mice (Figure 2F). There was no difference in the absolute number of β-cells between the two genotypes (Figure 2G). When we looked at insulin immunofluorescence staining signal intensity, we found that this was significantly lower in the *PolgA*^*mut/mut*^ mice at 44 weeks compared with *PolgA*^*+/+*^ mice (Figure 1A,I), suggesting that in the *PolgA*^*mut/mut*^ mice, complex I deficiency does not lead to absolute β-cell loss but does decrease insulin staining intensity.

To further understand the mechanistic basis underlying the change of cell composition in islets of 44 week *PolgA*^*mut/mut*^ mice, we examined islet cell composition changes within genotypes. In control *PolgA*^*+/+*^ mice, there was a significant increase in absolute β-cell number from 12 to 44 weeks (Supplementary Fig. 4D), leading to significant increases in whole islet cell number (Supplementary Fig. 4E) and islet size (Supplementary Fig. 4F). In *PolgA*^*mut/mut*^ mice, there were significant increases in the absolute numbers of both α- and β-cells from 12 to 44 weeks (Supplementary Figs. 4I and J), contributing to significant increases in whole islet cell number and islet size (Supplementary Fig. 4K and L). Quantification of the ratio of β-cells to α-cells at 44 weeks in both groups revealed a significantly lower β: α ratio in the islets of *PolgA*^*mut/mut*^ mice, compared with age-matched *PolgA*^*+/+*^ mice (3:1 vs 53:4, p < 0.01). Taken together, our data suggest that an increase in the number of α-cells accounted for the altered islet cell composition in 44 week *PolgA*^*mut/mut*^ mice.

### 3.3 Complex I expression is lower in α-cells compared with β-cells in islets from 44 week *PolgA*^*mut/mut*^ mice

**Figure 3:**
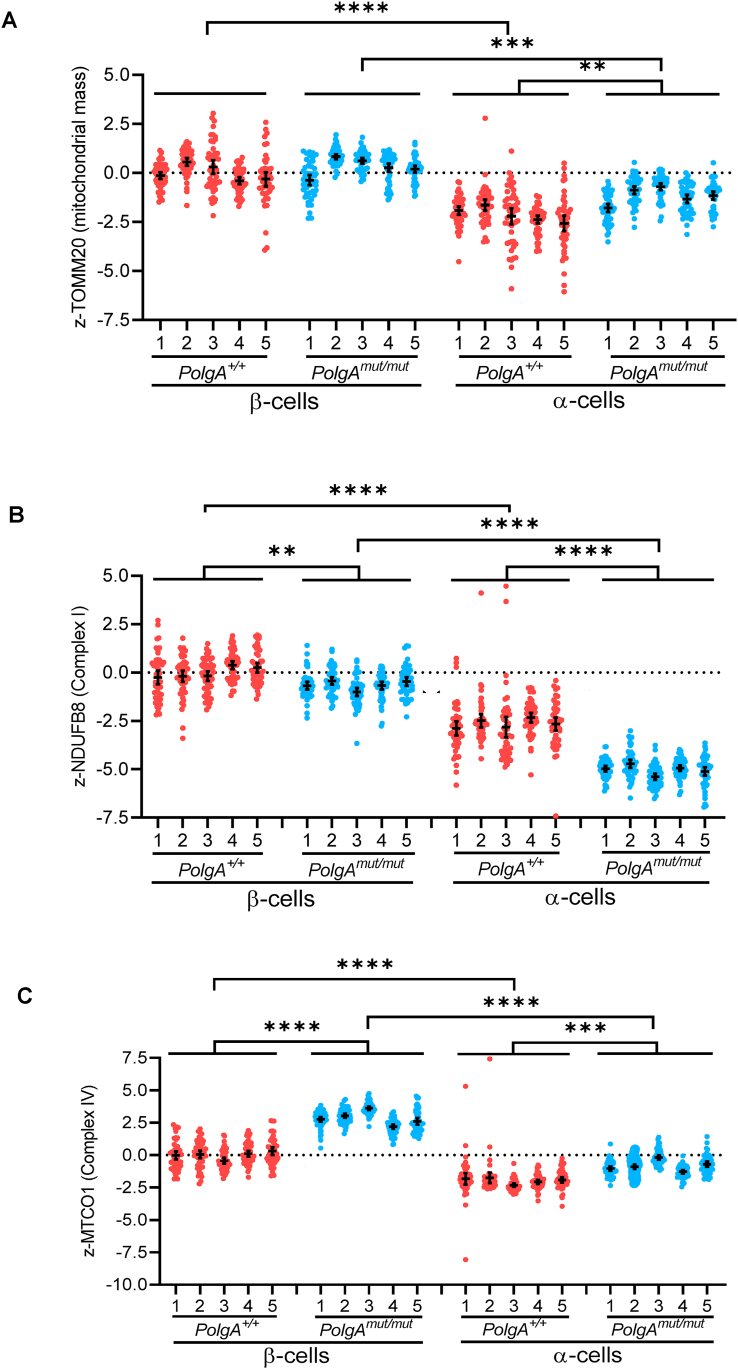
**Complex I expression is lower in α-cells compared with β-cells in islets from 44 week *PolgA*^*mut/mut*^ mice**. A–C: Quantification of levels of TOMM20 (A), NDUFB8 (B) and MTCO1 (C) in α-cells and β-cells in islets from 44 week *PolgA*^+/+^ (n = 5) and *PolgA*^*mut/mut*^ mice (n = 5). Data are presented as Z-scores relative to the *PolgA*^*+/+*^ β-cells. Each point represents an individual islet (n = 50 for each mouse). Data are presented as mean ± 95% CI. Unpaired t-test. ∗∗*P* < 0.01. ∗∗∗*P* < 0.001. ∗∗∗∗*P* < 0.0001.

In view of the increase in absolute α-cell number and altered islet cell composition in the 44 week *PolgA*^*mut/mut*^ mice, we next compared mitochondrial OXPHOS subunit expression in the populations of β- and non- β (predominantly α) cells in islets from 44 week *PolgA*^*mut/mut*^ mice and from age-matched *PolgA*^*+/+*^ controls (Figure 3). For comparative purposes, all data are presented as z-scores relative to the β-cells in the *PolgA*^*+/+*^ islets. Mitochondrial mass, complex I and complex IV protein levels were all significantly lower in the α-cells compared with the β-cells in both the *PolgA*^*+/+*^ and *PolgA*^*mut/mut*^ islets (Figure 3). Focussing on the individual cell types, there were significantly lower levels of complex I and higher levels of complex IV in α-cells from *PolgA*^*mut/mut*^ mice compared with α-cells from *PolgA*^*+/+*^ mice (Figure 3B,C). This was accompanied by a significant increase in mitochondrial mass (Figure 3A). A similar pattern was shown in the β-cells, though the decrease in complex I was less pronounced and the increase in complex IV was more pronounced in β-cells from *PolgA*^*mut/mut*^ mice compared with β-cells from *PolgA*^*+/+*^ mice (Figure 3B,C). Comparing the levels of complex I and IV protein subunits in α- and β-cells of *PolgA*^*mut/mut*^ mice, we found that complex I was significantly lower in the *PolgA*^*mut/mut*^ α-cells versus *PolgA*^*mut/mut*^ β-cells (p < 0.0001), and complex IV was significantly higher in the *PolgA*^*mut/mut*^ β-cells versus *PolgA*^*mut/mut*^ α-cells (p < 0.0001). No significant differences in the levels of the mitochondrial mass marker Tomm20were detected in the β-cells of the *PolgA*^*mut/mut*^ mice compared with *PolgA*^*+/+*^ controls (Figure 3A). These data show different patterns of mitochondrial subunit expression between pancreatic islet cell subtypes.

### 3.4 Increased α-cell proliferation in islets from 44 week *PolgA*^*mut/mut*^ mice

**Figure 4:**
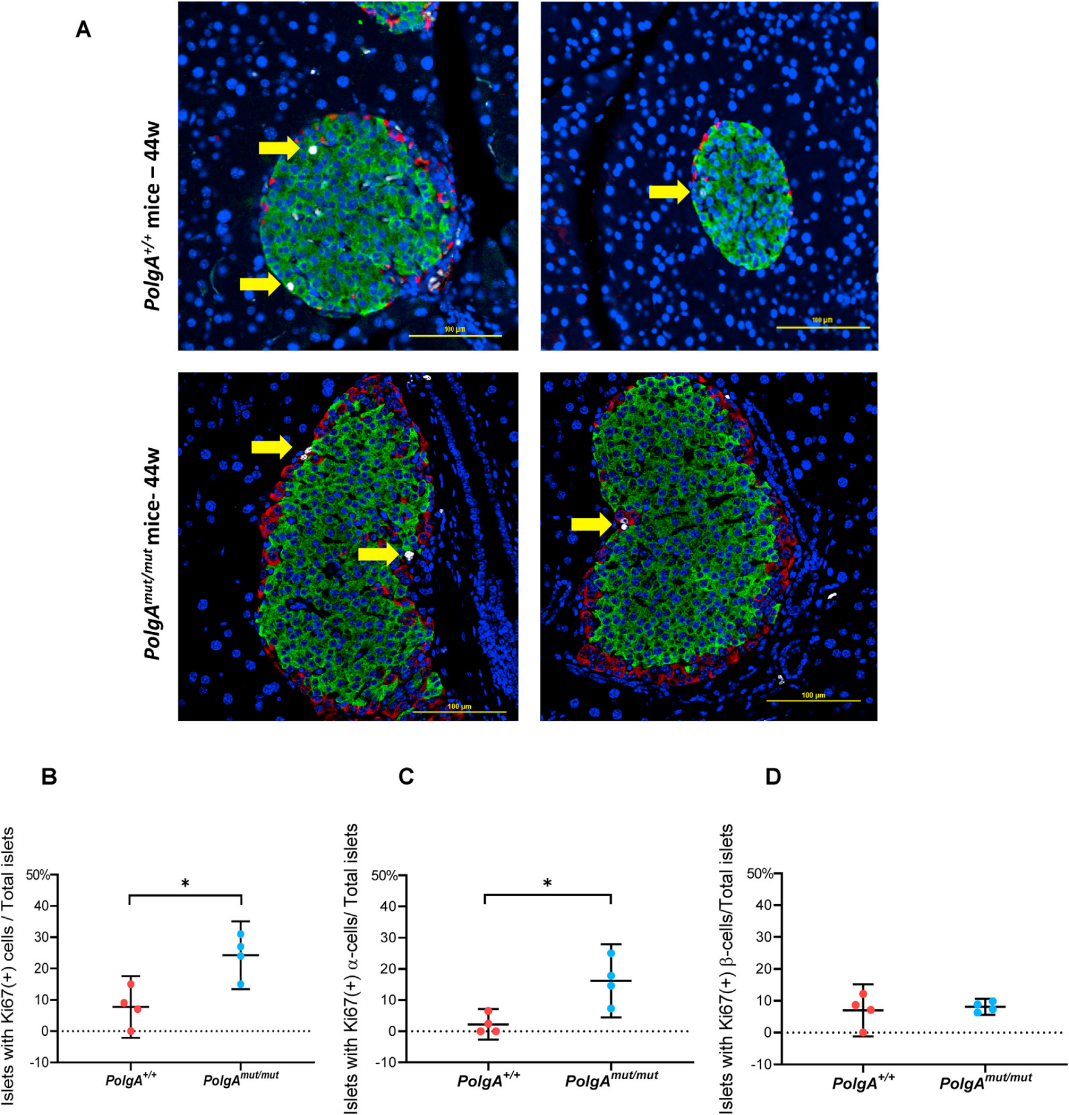
**Islets from 44 week *PolgA*^*mut/mut*^ mice show an increased frequency of proliferating α-cells compared with age matched controls** (A): Composite quadruple immunofluorescence panel showing labelling of; nuclei (DAPI, blue); proliferating cells (Ki67, white (highlighted by yellow arrows)); α-cells (Glucagon, red); and β-cells (Insulin, green) in pancreatic islets. (Top panel) *PolgA*^*+/+*^ mice; (Bottom panel) *PolgA*^*mut/mut*^ mice. Scale bar, 100 μm. (B–D): Quantification of Ki67 positive labelling in *PolgA*^*mut/mut*^ and *PolgA*^+/+^ mice. (B) Mean percentage of islets per mouse with ≥1 Ki67(+) cells. (C) Mean percentage of islets per mouse with ≥1 Ki67(+) α-cells. (D) Mean percentage of islets per mouse with ≥1 Ki67(+) β-cells. *PolgA*^+/+^ mice (n = 4); *PolgA*^*mut/mut*^ mice (n = 4). Each dot represents the mean percentage per mouse (40 islets were quantified per mouse). Data are presented as mean ± 95%CI. Unpaired t-test. ∗*P* < 0.05. (For interpretation of the references to color in this figure legend, the reader is referred to the Web version of this article.)

We next wanted to investigate the potential mechanisms underlying the changes in cell composition of pancreatic islets from 44 week *PolgA*^*mut/mut*^ mice. We first looked at levels of cell proliferation by immunofluorescence using the proliferation marker Ki67 (Figure 4A). The percentage of islets with one or more Ki67 positive stained cells was significantly higher in *PolgA*^*mut/mut*^ mice compared with age-matched controls (Figure 4B). When these data were stratified by cell type, this difference reflected a significantly higher percentage of islets with one or more Ki67 positive α-cells (Figure 4C), with no difference in the percentage of islets with one or more Ki67 positive β-cells (Fig. 4D). Analysing the data as the average number of Ki67 positive cells/islet gave the same results, with a significant increase in the number of Ki67 positive α-cells in the *PolgA*^*mut/mut*^ mice compared with age-matched controls (Supplementary Table 4). These data support an increase in α-cell proliferation in the islets from the *PolgA*^*mut/mut*^ mice.

We also looked for signs of other processes that might contribute to the altered cell composition in the islets from the *PolgA*^*mut/mut*^ mice. We searched for evidence of trans-differentiation by determining whether there was an increase in the number of bi-hormonal cells co-expressing both insulin and glucagon (representative images shown in Supplementary Fig. 5). The number of bi-hormonal cells expressed as a percentage of all the cells within an islet was comparable between the 44 week *PolgA*^*mut/mut*^ and age-matched *PolgA*^*+/+*^ control mice (0.40% ± 0.10% and 0.52% ± 0.13%, respectively). Similarly, the percentage of glucagon labelled cells within the β-cell core within each islet was comparable between the 44 week *PolgA*^*mut/mut*^ and age-matched *PolgA*^*+/+*^ mice (Supplementary Fig. 6). We next examined whether the relative change in the β-cell mass in the 44 week *PolgA*^*mut/mut*^ mice resulted from increased apoptosis. However, TUNEL analysis identified only one islet out of 50 *PolgA*^*mut/mut*^ mouse islets that harboured apoptotic cells, with none identified in the age-matched *PolgA*^*+/+*^ control islets (Supplementary Fig. 7).

### 3.5 *PolgA*^*mut/mut*^ mice have lower body weight and normal blood glucose levels compared with *PolgA*^*+/+*^ mice at 44 weeks

In view of the differences in pancreatic islet cell composition between the 44 week *PolgA*^*mut/mut*^ versus age-matched *PolgA*^*+/+*^ mice, we measured endpoint blood glucose concentration at the time of sacrifice. There was no significant difference in blood glucose level between the *PolgA*^*+/+*^ and *PolgA*^*mut/mut*^ mice (9.46 ± 1.17 mmol/l vs 8.9 ± 1.44 mmol/l, p > 0.05). These blood glucose level were all less than 13.3 mmol/l, the diagnostic cut off for diabetes [21]. However, there was a clear difference in endpoint body weight, with the 44 week control *PolgA*^*+/+*^ mice having significantly higher body weight than the age-matched *PolgA*^*mut/mut*^ mice (39.66 ± 6.06g vs 25.28 ± 3.64g, p < 0.01).

## 4 Discussion and conclusion

In this study we have found that decreased mitochondrial complex I protein expression leads to a significant decrease in β-cell insulin staining intensity and altered pancreatic islet cell composition in 44 week old *PolgA*^*mut/mut*^ mice. We show a significant increase in the proportion of α-cells per islet and provide evidence that is due to increased α-cell proliferation.

In the original description of the *PolgA*^*mut/mut*^ “mutator” mouse [16], it was found that young mice (8 weeks of age) already harboured a high mitochondrial mutation load. However, the features of the premature aging phenotype only started to appear after 25 weeks of age. By using a quantitative mitochondrial immunofluorescence assay [18], we were able to measure mitochondrial OXPHOS subunit levels within pancreatic islets for the first time. Our findings in the pancreatic islets are consistent with Trifnovic et al.'s findings in other tissues [16], with evidence of a marked decrease in complex I expression in islets from 12 week *PolgA*^*mut/mut*^ mice but with no change in islet cell composition or insulin staining intensity. The impact of decreased complex I subunit expression only became apparent in the 44 week old animals.

It has been previously shown that β-cell-specific knock-out (KO) of TFAM, which is essential for mtDNA expression and maintenance, results in severe mtDNA depletion, defects in OXPHOS and development of diabetes by the age of 5 weeks [15]. The authors reported a loss of complex IV enzyme activity and lowered insulin secretion at 7–8 weeks of age, followed by a significant decrease in the islet β:α cell ratio at 39 weeks of age. While this change in the islet β:α cell ratio was comparable to that seen in the 44 week old *PolgA*^*mut/mut*^ islets in our study, β-cell loss was a major driver of the altered islet cell composition in TFAM KO mice. This model has a much more severe OXPHOS deficient phenotype in the islets compared with the *PolgA*^*mut/mut*^ mice, hence causing the earlier onset of impaired insulin secretion. The TFAM KO mice model also showed an apparent recovery of complex IV activity in islets of the older mice. This was thought to be due to a loss of the complex IV deficient β-cells with some compensatory replacement with β-cells which had escaped cre-recombination and had normal complex IV activity [15]. Interestingly, in our study we also saw a specific increase in complex IV protein levels in both α-cells and β-cells at 44 weeks of age along with a decrease in insulin expression, suggesting that some level of complex IV function is critical for islet function. It is possible that the increased complex IV expression represents an adaptive response to the mitochondrial stress in the pancreatic β-cells in the *PolgA*^*mut/mut*^ mice. It has been shown in patients with inherited mtDNA mutations that a defect in one or more OXPHOS subunits can induce compensatory mitochondrial biogenesis [22], and a similar mechanism may explain the increase in mitochondrial mass observed between 12 and 44 weeks in the *PolgA*^*mut/mut*^ pancreatic islets. This might also be the reason why we do not see the decrease in complex IV subunit levels in the 44 week *PolgA*^*mut/mut*^ mice, contrary to that seen in the islets of the 44 week *PolgA*^*+/+*^ mice. It could be that the compensatory increase in total mitochondrial mass in the islets harbouring complex I deficiency maintains complex IV levels over time. There was no investigation of complex I function in the β-cell-specific TFAM knockout islets. However, other studies have shown reduced expression of complex I subunits and reduced complex I activity in TFAM knockout mice [23], and therefore complex I would be predicted to also be deficient in the TFAM model. Although we did not see any deficiency in complex IV protein levels in the islets, we were not able to measure complex IV activity in the current study using fixed tissue, therefore it's difficult to rule out that there were alterations in the enzyme activity.

A novel and striking finding is the increase in α-cell number in the *PolgA*^*mut/mut*^ islets, which resulted in a significantly decreased β: α cell ratio in islets from the 44 week *PolgA*^*mut/mut*^ mice compared with the age-matched controls. Examination of Ki67 expression showed evidence of increased α-cell proliferation in 44 week *PolgA*^*mut/mut*^ islets. Lam and colleagues have provided evidence that α-cells in islets from healthy mice primarily expand by self-renewal (i.e proliferation of existing α-cells) rather than from specialized progenitors [24].

There are a number of potential mechanisms that could explain the increased α-cell number. There was marked complex I deficiency in the α-cells of 44 week *PolgA*^*mut/mut*^ mice compared with the β-cells of the 44 week *PolgA*^*mut/mut*^ mice. Knockdown of NDUFB9, a component of complex I, has been shown to promote cell proliferation in a tumour cell-line and was accompanied by an increase in reactive oxygen species (ROS) generation [25], and raised ROS levels have been implicated in promoting cell proliferation in non-tumour cell lines [26]. Using the same *PolgA*^*mut/mut*^ mouse model, examination of mitochondrial ROS generation *in vivo* showed that ROS levels were normal in young mice despite marked mitochondrial dysfunction, but increased as the animals aged [27]. Together, these findings raise the possibility that the increased α-cell proliferation observed in 44 week *PolgA*^*mut/mut*^ mouse islets is a direct response to complex I deficiency and increased ROS generation. Alternatively, increased α-cell proliferation could arise secondarily to dysfunction in other cell types. For example, streptozotocin induced diabetes in healthy mice was accompanied by decreased insulin and GABA expression in β-cells and an increase in α-cell number. GABA treatment reversed the α-cell hyperplasia pointing to a link between β-cell function and α-cell mass [28]. While GABA expression was not measured in this study, we did find evidence of decreased insulin staining intensity consistent with impaired β-cell function in the pancreatic islets of the 44 week *PolgA*^*mut/mut*^ mice.

Other mechanisms might contribute to the lower β: α cell ratio in islets from the 44 week *PolgA*^*mut/mut*^ mice, including trans-differentiation of β-to α-cells [29]. We postulated that if this process was a key factor, we would expect to see more glucagon staining cells within the insulin staining inner β-cell core and more bi-hormonal staining cells in the 44 week *PolgA*^*mut/mut*^ islets. This turned out not to be the case, suggesting that trans-differentiation is unlikely to be a major contributor to the increased α-cell number in the 44 week *PolgA*^*mut/mut*^ islets. Furthermore, we found no evidence of wide-scale altered apoptosis in the *PolgA*^*mut/mut*^ islets, in keeping with observations from islets of the TFAM KO mouse [15].

Li and colleagues observed a diminished insulin response *in vivo* and impaired glucose tolerance in aged *PolgA*^*mut/mut*^ mice, but found no change in whole body insulin sensitivity [30]. In our study, the decreased insulin staining intensity and increased α-cell mass in the islets of 44 week *PolgA*^*mut/mut*^ mice would be predicted to promote abnormal glucose intolerance. However, we found no difference in end-point glucose concentrations between 44 week *PolgA*^*mut/mut*^ mice and age-matched controls. This may be because mean body weight was markedly decreased in the 44 week *PolgA*^*mut/mut*^ mice, which would decrease the insulin secretory demand made upon the islets and help to maintain normal glucose homeostasis. Our findings are in keeping with the original description of the aged *PolgA*^*mut/mut*^ mouse in which body weight was decreased due to a specific decrease in whole body adiposity [16].

Taken together, our findings show that complex I subunit deficiency adversely affects pancreatic β-cell function and islet cell composition. These changes would be predicted to predispose to abnormal glucose tolerance, particularly under conditions of increased insulin resistance and increased secretory demands placed on the pancreatic β-cell cells.

## Author contributions

Conceptualization, M.W. and L.G.; Methodology, X.Y., L.G., M.W., C.B., A.L.M.S., J.W., R.B.P., N.K., C.A., C.C., S.A., M.W., J.W., D.T., J.S.; Investigation X.Y., M.W., L.G.; Formal Analysis, X.Y. C.C., R.B.P.; Supervision and Fund Acquisition, M.W L.G.; Writing – Review & Editing, X.Y., M.W., L.G., J.S., D.T., C.A.; M.W is the guarantor of this work and have full access to all data in the study and takes full responsibility for the integrity of the data and the accuracy of the data analysis.

## Prior presentation information

A part of this work has been presented following peer-review to the international mitochondrial, diabetes and islet communities at the 54th European Association for the Study of Diabetes (2018) and 79th scientific session of American Diabetes Association (2019) annual meetings.

## Funding

This work was supported by the 10.13039/501100013372Wellcome Trust Centre for Mitochondrial Research [203105/Z/16/Z], Newcastle University Centre for Ageing and Vitality (supported by the Biotechnology and Biological Sciences Research Council, Engineering and Physical Sciences Research Council, Economic and Social Research Council and Medical Research Council [MR/L016354/1]), and a Newcastle University Oversea Research Scholarship.
