# Supplementary material for: Mitochondrial complex I subunit deficiency promotes pancreatic α-cell proliferation
Source: Mol Metab. 2022 Apr 4;60:101489. doi: 10.1016/j.molmet.2022.101489 (PMC9046450; doi:10.1016/j.molmet.2022.101489)
Supplement: Multimedia component 1 [file mmc1.docx]

**Supplemental material**

**Figure 1**


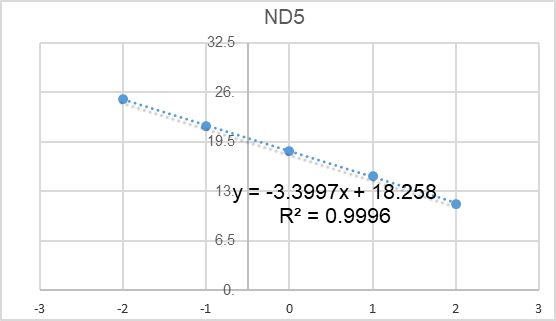

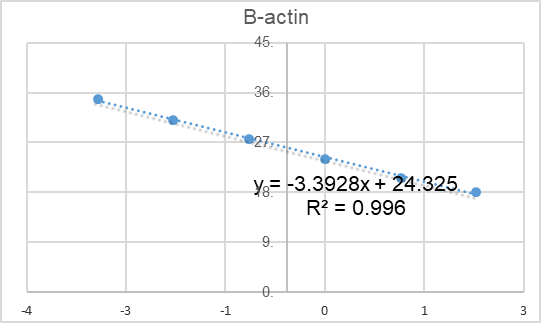


**A B**

**C**

**D**

**Figure S1. No significant difference in islet cell mtDNA copy number between 44 week *PolgA^mut/mut^* mice and age-matched *PolgA^+/+^* mice. Relate to Figure 1.**

**(A) Standard curve of ND5 target gene prepared using a series of 1 in 10 dilutions of *PolgA^+/+^* mouse pancreas DNA*.*** The amplification curve starts from the first dilution of ND5 (concentration 100 ng/µl) and ends with the fifth dilution (concentration 1×10^-2^ ng/µl). ND5: R^2^: 0.9996. Slope: -3.3997. Y-intercept: 18.258.

**(B) Standard curve of B-actin reference gene prepared using a series of 1 in 10 dilutions of *PolgA^+/+^* mouse pancreas DNA*.*** The amplification curve starts from the first dilution of B-actin (concentration 100 ng/µl) and ends with the sixth dilution (concentration 1×10^-3^ ng/µl). B-actin: R^2^: 0.996. Slope: -3.3928. Y-intercept: 24.325.

**(C-D) Pancreatic islet mtDNA copy number comparison between 44 week *PolgA^mut/mut^* mice and age-matched *PolgA^+/+^* mice.** (C) Standard curve analysis method. (D) 2*(2^ (-∆Ct)) analysis method. Each dot represents one islet (n=25 for each group). Data are presented as mean ± 95% CI. Unpaired t-test.

**Research methods for results shown in Figure S1**

**Laser Dissection**

For individual islet DNA extraction, frozen pancreas sections were subjected to frozen section immunofluorescence as above to label glucagon in order to locate the whole islet. Tiled images were captured by Zen Blue in Axio Imager M1 microscope (Carl Zeiss) in order to map individual islets on pancreas frozen sections. Labelled sections were prepared for laser microdissection by dehydrating through an ascending ethanol gradient (70%, 95%, 100%, and 100%, 10 minutes each) and placed in a sterile hood for half an hour. The islets were outlined based on glucagon signal in the 568 nm channel and laser-captured using the PALM laser micro-dissection system (Zeiss) as described in [18]. For each section, over 10 islets were captured and collected individually in 10 μl of Tris-Tween lysis buffer (0.5 M Tris- HCl, 0.5% Tween- 20, 1% Proteinase K, pH 8.5). Single cells were lysed for 2 h at 55 °C, followed by 10 min at 95 °C. This part of results are reported in **Figure S1**.

**Real Time PCR**

Following extraction from dissected islet samples, real-time PCR reaction was conducted on a LightCycler 480 detector by using SYBR green (Invitrogen). DNA sample was amplified using either ND5 or β-actin primers. Standard curves of ND5 target gene and β-actin reference gene prepared using a series of five 1 in 10 dilutions of wild type mouse pancreas DNA (from 100 to 0.01 ng/ul). Standard curve analysis and delta Ct quantification were performed to calculate the mtDNA copy number. This part of results are reported in **Figure S1**.

**Figure 2**

**A B C**

**D E F**

**Figure S2. Quantification of the levels of OXPHOS proteins in 12 and 44 week *PolgA^+/+^* and *PolgA^mut/mut^* mice. Related to Figure 1. (A-C):** Quantitative immunofluorescent analysis of TOMM20 (**A**), NDUFB8 (**B**) and MTCO1 (**C**) in islets from 12 and 44 week *PolgA^+/+^* mice (n=4 mice per group, n=25 islets quantified per mouse). Data are presented as z-scores relative to the 12 week *PolgA^+/+^* mice. **(D-F):** Quantitative immunofluorescent analysis of TOMM20 (**D**), NDUFB8 (**E**) and MTCO1 (**F**) in islets from 12 and 44 week *PolgA^mut/mut^* mice (n=4 mice per group, n=25 islets quantified per mouse). Data are presented as z-scores relative to the 12 week *PolgA^mut/mut^* mice. For all panels each dot represents an individual islet. Error bars show mean ± 95% CI**.** Unpaired t-test. ***P* < 0.01.

**Figure 3**

**A**


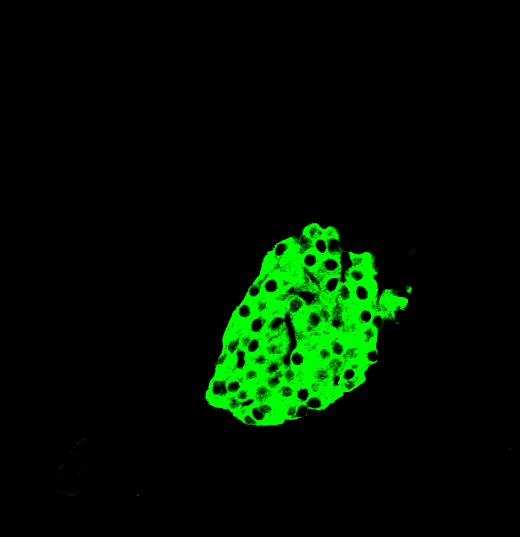

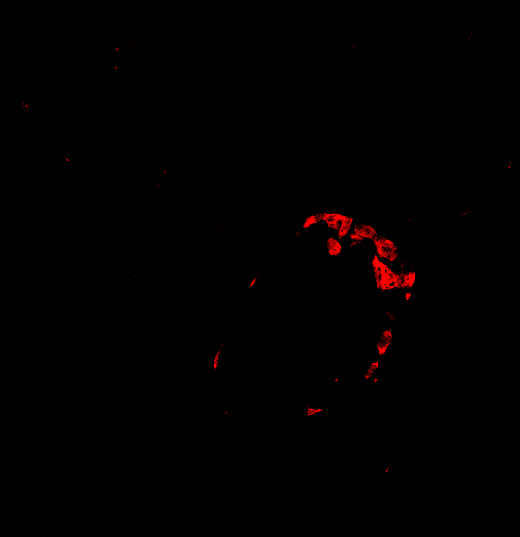

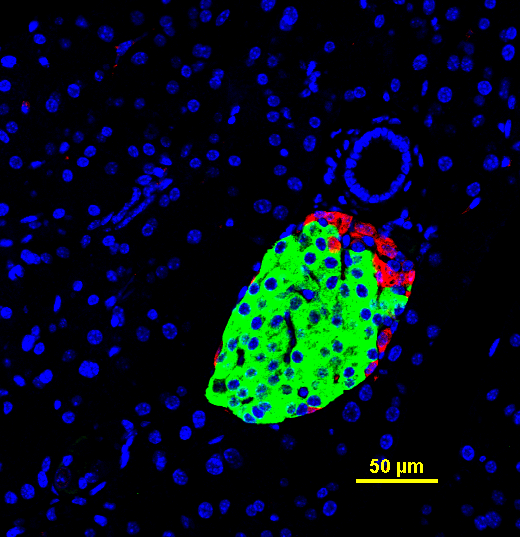

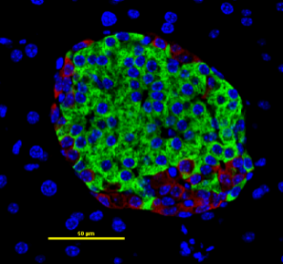

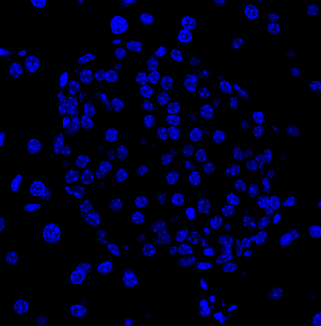

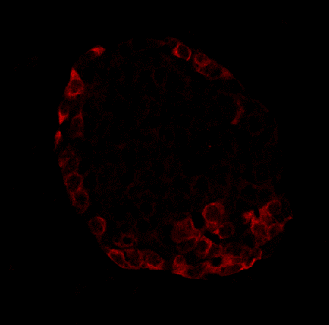

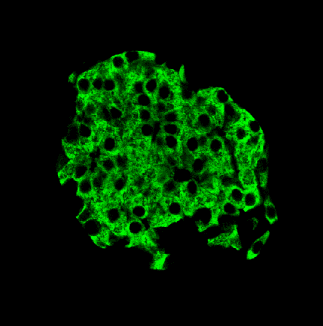


**Composite Nucleus Glucagon Insulin**

***PolgA^mut/mut^*-12w *PolgA^+/+^*-12W**


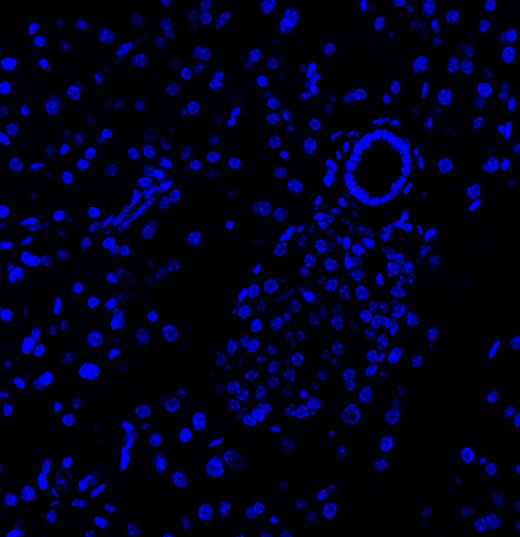

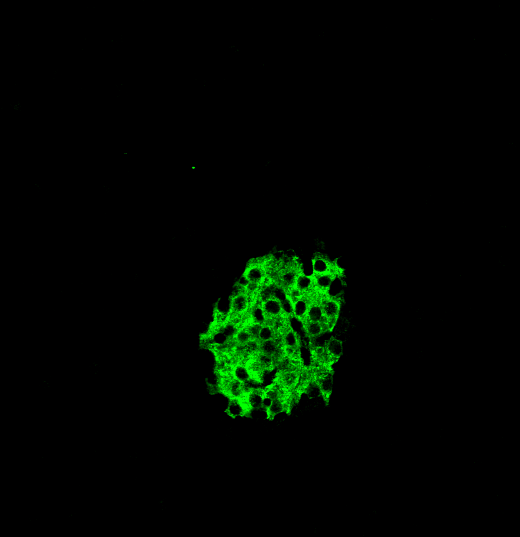

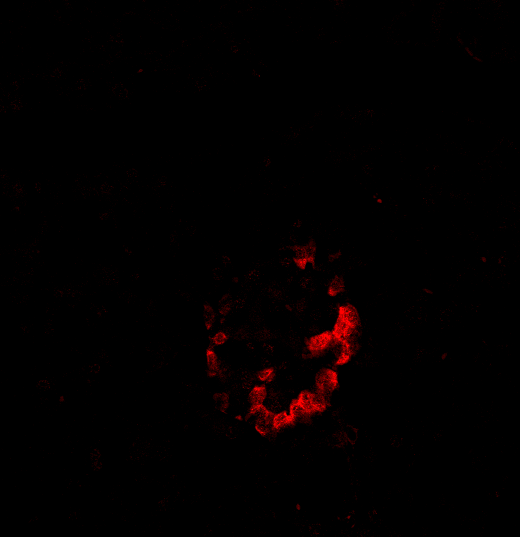

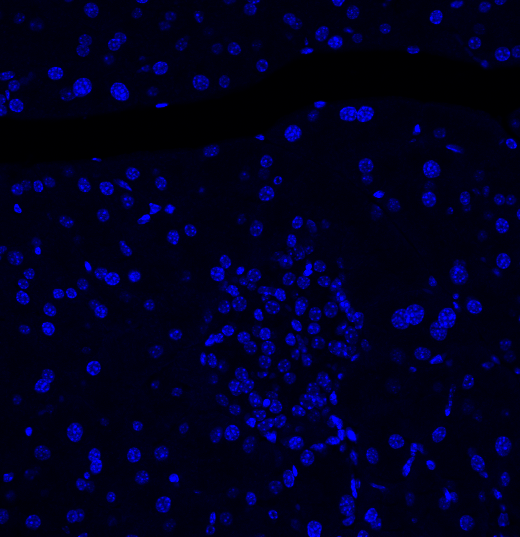

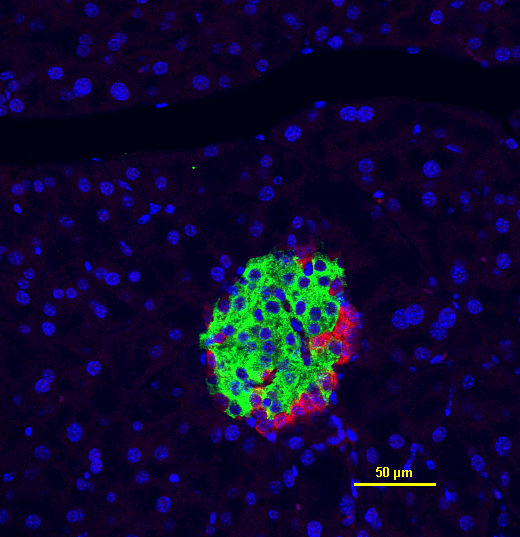

**B C D**

**E F G**

**Figure S3. There were no significant differences in the α- and β-cell percentage, islet size and islet cell number between 12 week *PolgA^mut/mut^* mice and age-matched *PolgA^+/+^* mice. Related to Figure 2.**

**(A)** Immunofluorescent panel showing labelling of endocrine hormones in pancreatic islets from 12 week *PolgA^+/+^* and *PolgA^mut/mut^* mice. From left to right, immunofluorescence labelling: composite; nuclei (DAPI); α-cells (Glucagon); β-cells (Insulin). Scale bar, 100µm.

**(B-G) Islet cell composition of 12 week *PolgA^+/+^* and *PolgA^mut/mut^* mice. (B)** α cell percentage. **(C)** β cell percentage. **(D)** Islet size. **(E)** Whole islet cell number. **(F)** Absolute α-cell number. **(G)** Absolute β-cell number. *PolgA^+/+^* mice (Left 1-4, n=4); *PolgA^mut/mut^* mice (Right 1-4, n=4).

Each point represents one islet (n=25 for each mouse). Data are presented as mean ± 95% CI. Unpaired t-test.

**Figure 4**

**A B C**

**D E F**

**G H I**

**J K L**

**Figure S4. The age-related islet cell composition changes within genotypes. Related to Figure 2.**

**(A-F) Absolute β-cell number, islet size and whole islet cell number all increased significantly in *PolgA^+/+^* mice with age. (A)** α cell percentage. **(B)** β cell percentage. **(C)** Absolute α-cell number. **(D)** Absolute β-cell number. **(E)** Whole islet cell number. **(F)** Islet size. 12 week *PolgA^+/+^* mice (Left 1-4, n=4); 44 week *PolgA^+/+^* mice (Right 1-4, n=4).

**(G-L) Absolute α- and β-cell number, islet size and whole islet cell number all increased significantly in *PolgA^mut/mut^* mice with age. (G)** α cell percentage. **(H)** β cell percentage. **(I)** Absolute α-cell number. **(J)** Absolute β-cell number. **(K)** Whole islet cell number. **(L)** Islet size. 12 week *PolgA^mut/mut^* mice (Left 1-4, n=4); 44 week *PolgA^mut/mut^* mice (Right 1-5, n=5).

Each dot represents an individual islet (n=25 for each mouse). Data are presented as mean ± 95%CI**.** Unpaired t-test. **P* < 0.05. ***P* < 0.01. ****P* < 0.001.

**Figure 5**

**Nucleus Insulin Glucagon Composite**

**
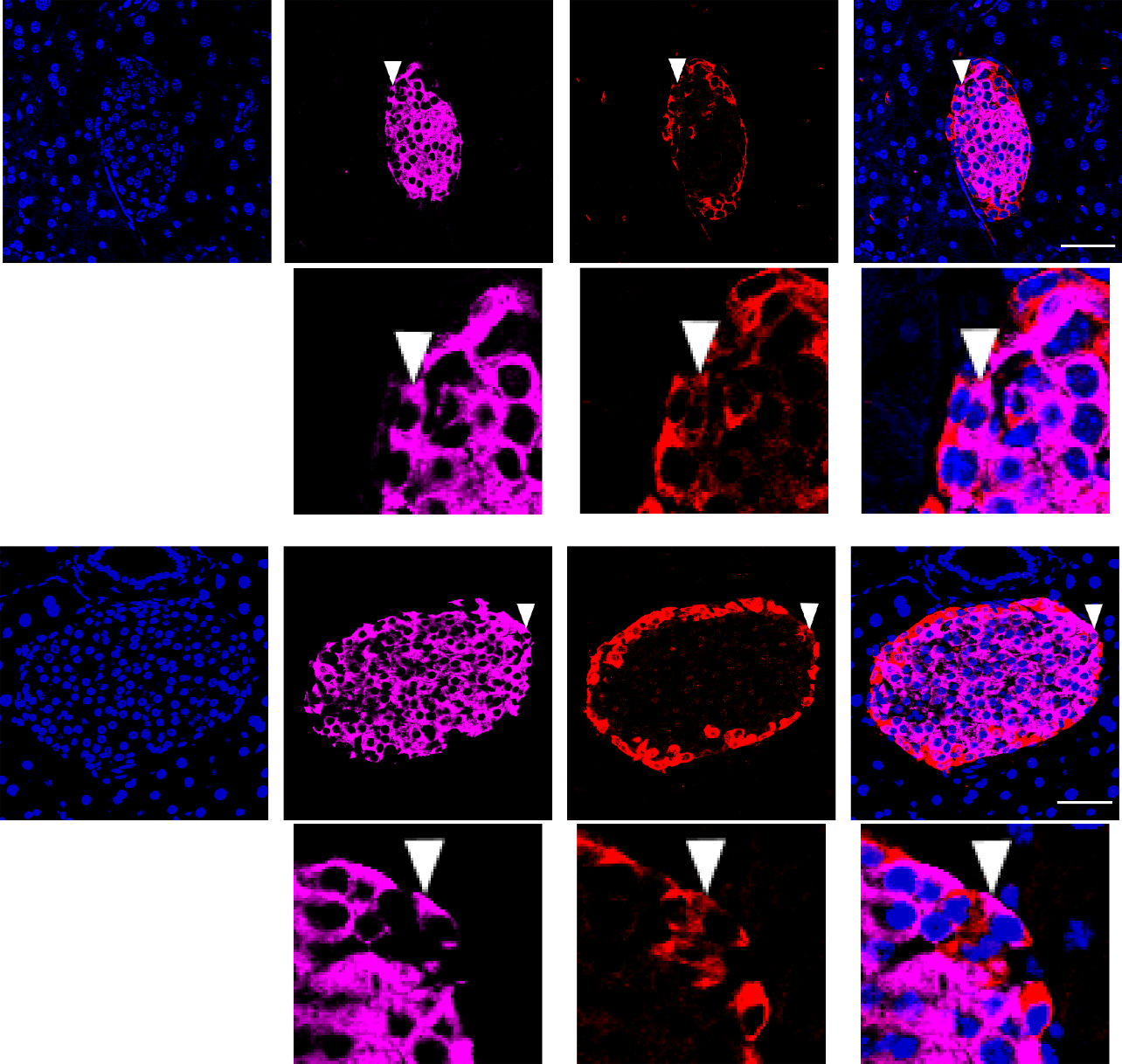
**

**Zoom Zoom**

***PolgA^mut/mut^*-44w *PolgA^+/+^*-44W**

**Figure S5. There were no significant differences in the number of bihormonal (glucagon and insulin positive) cells between 44 week *PolgA^mut/mut^* mice and the age-matched *PolgA^+/+^* mice.**

**Immunofluorescence panel showing labelling of endocrine hormones in pancreatic islets from 44 week mice.** (Top row) *PolgA^+/+^* mice; (Bottom row) *PolgA^mut/mut^* mice. From left to right, immunofluorescence labeling: nuclei (DAPI); Insulin, Glucagon, Composite. Scale bar, 50µm. Arrows indicate bihormonal cell. Image is representative of at least 9 islets from 4 *PolgA^mut/mut^* or 4 *PolgA^+/+^* mice.

**Figure 6**


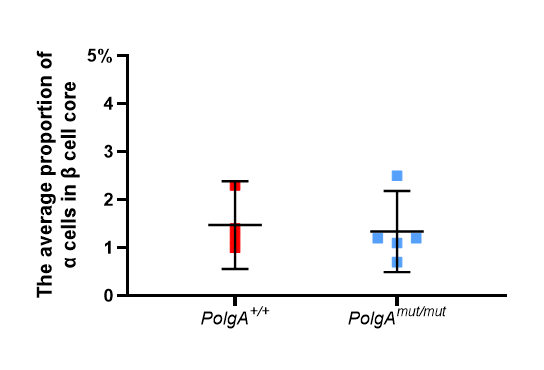


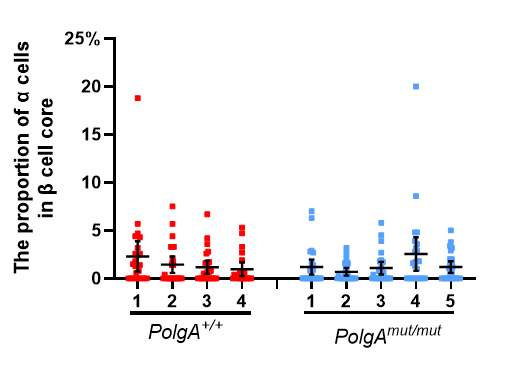


**A B**

**Figure S6. There were no significant differences in the proportion of α-cells in β-cell core between 44 week *PolgA^mut/mut^* and the age-matched *PolgA^+/+^* islets.**

**(A) The summary of α-cell proportion in β-cell core in islets from 44 week *PolgA^mut/mut^* mice and age-matched *PolgA^+/+^* mice.** *PolgA^+/+^* mice (Left 1-4, n=4); *PolgA^mut/mut^* mice (Right 1-5, n=5). Each dot represents an individual islet (n=25 for each mouse). Data are presented as mean ± 95%CI and analyzed by unpaired t-test.

**(B) The average summary of α-cell proportion in β-cell core in islets from 44 week *PolgA^mut/mut^* mice and age-matched *PolgA^+/+^* mice.** Each dot represents average data of 25 islets from one mouse. Data are presented as mean ± 95%CI and analyzed by unpaired t-test.

**Figure 7**


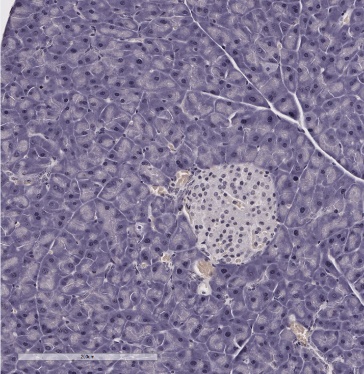

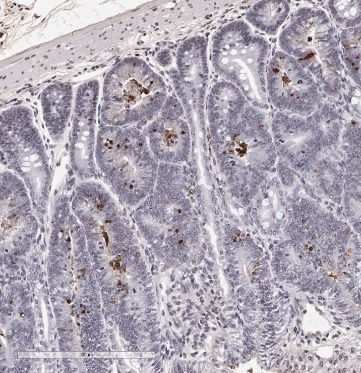

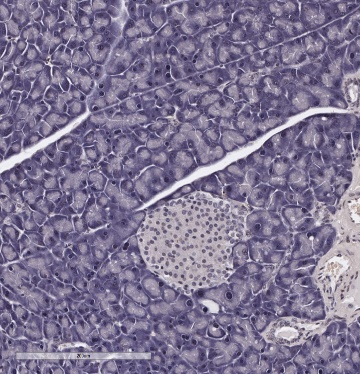

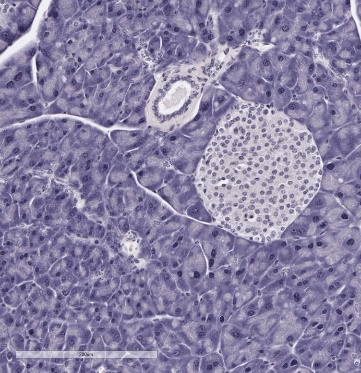


**A B**

**C D**

**Figure S7. TUNEL assay to detect apoptotic cells in islets from 44 week *PolgA^mut/mut^* and age-matched *PolgA^+/+^* mice.** Scale bar, 200µm.

1. **Negative control for the TUNEL assay.** The enzyme solution has been omitted from the assay. No apoptotic nuclei were detected in *PolgA^mut/mut^* islet.
2. **Positive control for the TUNEL assay.** Mouse small intestinal adenoma tissue was used and apoptotic nuclei identified by the brown staining.
3. ***PolgA^mut/mut^* islet stained with TUNEL assay.** No apoptotic nuclei were detected.
4. ***PolgA^+/+^* islet stained with TUNEL assay.** No apoptotic nuclei were detected.

**Table 1. Detailed information of mice used in this study.**

| Harvest number | Genotype | Gender | Age |
| --- | --- | --- | --- |
| PolG1622 | *PolgA^+/+^* | Male | 12 weeks |
| PolG1624 | *PolgA^+/+^* | Male | 12 weeks |
| PolG1626 | *PolgA^+/+^* | Male | 12 weeks |
| PolG1629 | *PolgA^+/+^* | Male | 12 weeks |
| PolG1681 | *PolgA^mut/mut^* | Male | 12 weeks |
| PolG1682 | *PolgA^mut/mut^* | Male | 12 weeks |
| PolG1683 | *PolgA^mut/mut^* | Male | 12 weeks |
| PolG1709 | *PolgA^mut/mut^* | Male | 12 weeks |
| GA0002 | *PolgA^+/+^* | Male | 44 weeks |
| GA0004 | *PolgA^+/+^* | Male | 44 weeks |
| GA0005 | *PolgA^+/+^* | Male | 44 weeks |
| GA0007 | *PolgA^+/+^* | Male | 44 weeks |
| GA0008 | *PolgA^+/+^* | Male | 44 weeks |
| LG0002 | *PolgA^mut/mut^* | Male | 44 weeks |
| LG0004 | *PolgA^mut/mut^* | Male | 44 weeks |
| LG0005 | *PolgA^mut/mut^* | Male | 44 weeks |
| LG0006 | *PolgA^mut/mut^* | Male | 44 weeks |
| LG0011 | *PolgA^mut/mut^* | Male | 44 weeks |
| LG0024 | *PolgA^mut/mut^* | Male | 44 weeks |
| LG0032 | *PolgA^mut/mut^* | Male | 44 weeks |
| LG0036 | *PolgA^mut/mut^* | Male | 44 weeks |
| LG0037 | *PolgA^mut/mut^* | Male | 44 weeks |
| Polg1909 | *PolgA^mut/mut^* | Female | 44 weeks |
| Polg1918 | *PolgA^mut/mut^* | Male | 44 weeks |
| Polg1923 | *PolgA^mut/mut^* | Female | 44 weeks |
| Polg1937 | *PolgA^mut/mut^* | Male | 44 weeks |
| Polg1940 | *PolgA^mut/mut^* | Female | 44 weeks |
| Polg1967 | *PolgA^+/+^* | Male | 44 weeks |
| Polg1997 | *PolgA^+/+^* | Male | 44 weeks |
| Polg1999 | *PolgA^+/+^* | Male | 44 weeks |
| Polg2029 | *PolgA^+/+^* | Female | 44 weeks |
| Polg2034 | *PolgA^+/+^* | Male | 44 weeks |

**Table 2. Information for primary antibodies used in our study.**

| Category | Primary Antibody | Species raised in | Clonality | Isotype | Confirmed specificity | Dilution | Supplier & catalogue number |
| --- | --- | --- | --- | --- | --- | --- | --- |
| Mitochondrial primary antibodies | Insulin | Guinea pig | Polyclonal |  | Human, Mouse, Rat | 1:200 | DAKO  IR00261-2 |
|  | Tomm20 | Rabbit | monoclonal | IgG | Mouse, Rat, Human | 1:100 | Abcam  ab186734 |
|  | MTCO1 | Mouse | monoclonal | IgG2a | Mouse, Rat, Goat, Cow, Human | 1:100 | Abcam  ab14705 |
|  | NDUFB8 | Mouse | monoclonal | IgG1 | Mouse, Rat, Cow, Human, Pig | 1:100 | Abcam  ab110242 |
|  | | | | | | | |
| Pancreatic hormone primary antibodies | Insulin | Guinea pig | Polyclonal |  | Human, Mouse, Rat | 1:200 | DAKO  IR00261-2 |
|  | Glucagon | Mouse | Monoclonal | IgG1 | Rat, Pig, Guinea pig, Mouse, Canine, Human, Rabbit | 1:200 (1:100 for human) | Sigma  G2654 |
|  | Ki67 | Rabbit | Monoclonal | IgG | Human, Mouse, Rat | 1:400 | Cell signaling  D3B5 |

**Table 3. Information for secondary antibodies used in our study.**

| Category | Species raised in and isotype | Antibody against | Conjugation | Dilution | Supplier and catalogue number | Corresponding primary antibody |
| --- | --- | --- | --- | --- | --- | --- |
| Mitochondrial secondary antibodies | Guinea pig IgG | Goat | DAPI | 1:200 | Abcam  ab175678 | Insulin |
|  | Rabbit IgG | Goat | FITC | 1:200 | Invitrogen A11008 | Tomm20 |
|  | Mouse IgG2a | Goat | TRITC | 1:200 | Invitrogen A21133 | MTCO1 |
|  | Mouse IgG1 | Goat | Biotin | 1:200 | Invitrogen A10519 | NDUFB8 |
|  |  |  | Streptavidin Alexa 647 | 1:100 | Invitrogen  S32357 |  |
|  | | | | | |  |
| Pancreatic hormone secondary antibodies | Rabbit IgG1 | Goat | FITC | 1:500 | Invitrogen A11008 | Ki67  Glucagon  Insulin |
|  | Mouse IgG | Donkey | TRITC | 1:250 | Life Technologies  A10037 |  |
|  | Guinea pig IgG | Goat | Alexa 647 | 1:250 | Life Technologies  A21450 |  |

**Table 4. The number of Ki67 (+) islets in each mouse.**

| Case number | Genotype | Total number of Ki67(+) cells | Total number of Ki67 (+) α-cells | Total number of Ki67 (+) β-cells | No of islets counted |
| --- | --- | --- | --- | --- | --- |
| GA0004 | *PolgA^+/+^* | 6 | 1 | 5 | 41 |
| GA0005 | *PolgA^+/+^* | 4 | 0 | 4 | 42 |
| GA0007 | *PolgA^+/+^* | 0 | 0 | 0 | 42 |
| GA0008 | *PolgA^+/+^* | 9 | 3 | 6 | 46 |
| LG0005 | *PolgA^mut/mut^* | 25 | 21 | 4 | 64 |
| LG0024 | *PolgA^mut/mut^* | 12 | 8 | 4 | 41 |
| LG0032 | *PolgA^mut/mut^* | 6 | 3 | 3 | 41 |
| LG0037 | *PolgA^mut/mut^* | 13 | 9 | 4 | 45 |

The average number of Ki67 positive alpha cells/islet was higher for the *PolgA^mut/mut^* versus *PolgA^+/+^*control mice (median[IQR]: 0.198[0.104-0.294] vs 0.012[0-0.055]; p=0.03), with no difference for Ki67 positive beta-cells/islet (0.081[0.067-0.095] and 0.109[0.024-0.128]; p=0.47)
